# Ars Longa

**Published:** 1893-12-30

**Authors:** 


### The Hospital

AN INSTITUTIONAL JOURNAL OF

Science, Medicine, Hygiene, Nursing, and News.

*For Hospitals, Asylums, Infirmaries, Dispensaries, Medical Practitioners, Students, and Nurses.*

VOL. XV.—No. 379.] [DEC. 30, 1893.

### ARS LONGA.

THE close of another year reminds us not only that life is short, but still more that "art is long." In no previous year within the writer's recollection has "quackery" ever been so rife, or so much in evidence. What is quackery: and why do people love it so much? It is an attempt to seize by a bold fluke and *per saltum* what can only be acquired by steady and painstaking labour. People love quackery because it promises them a result which is manifestly remote without insisting upon their making the long and arduous journey which appears to be the only other way of arriving at the result. In other words, quackery is the exact antipodes of art. The crude person never possesses the artistic capacity: the crude person is therefore the easy victim of the quack. Quackery and crudity are fox and goose: the one is made juicy in order that the other may dine sumptuously.

Sickness lends itself to quackery as hunger lends itself to theft. The sick person suffers and is impatient. The art of scientific medicine is necessarily sometimes gradual in the cure. Impatient sickness grasps the hand of the quack because he promises to give at once what true art declares can only come by slow degrees. The hungry man, likewise, because he feels the keenness of hunger's pangs, will listen to the voice within which tells him of the pleasure of eating and of the harmlessness of theft. We do not say it is as great a moral offence to consult a quack as to steal a leg of mutton; but we do say that it is as foolish, and as likely to lead to disastrous results. The thief eats his mutton; and then goes to prison for a month to feed on bread and water: similarly the victim of the quack despises the slow but truly progressive medical art, and usually finds himself travelling in a disappointing circle of interminable but unfulfilled promises.

But it is far from our present purpose to write a disquisition on quackery. The medicine of the future is like a great mountain which science must level to a plain; or like a vast arm of the sea between two neighbouring parts of the same country which victorious art must bridge over. Nay, it is even more difficult still. There is no problem so hard of solution as that of bringing in universal health; unless, indeed, it be that other world-enduring problem of bringing in univeral virtue. To the healthy man health is easy, as virtue is to the virtuous. But the deviations from the normal standard of health and virtue are so manifold, so various, and often so antagonistic, that the two problems seem well-nigh insoluble. Well may the physician echo the utterance that "art is long;" and well may he be indignant at the impudent pretender who, without any capacity for art at all, promises the commencement of the golden age of health for a money payment.

Until true science dawned men never dreamt how infinitely complex an organism the human body is. "What a strange thing it is," said a patient the other day, "that I should be so frequently troubled with this tiresome sore throat." "Say rather," answered his doctor, "what a much stranger thing it is that you are not troubled with continuous sore throats, and with ten thousand other ills at the same time." The wonder, to the philosophical observer is, not that some people are sometimes ill, but that any of the innumerable people in the world are ever well. The human body, if it were a mere non-living machine, would be a standing miracle, merely because in spite of its indescribable complexity it continues to hold together for seventy or eighty years. But when we consider the infinite variety of physical, mental, chemical, and vital work it does during that period, and still holds together, well may we exclaim that "a miracle and a wonder unsurpassed is presented for our profoundest thoughts."

If we begin at the nervous system, with its capacity for receiving impressions and giving reflex results; with its power of sensation, of conversion, of transference, of motion; with its special organs for sight, for hearing, for taste, for smell; with its controlling authority over every part of the general organism; over the blood stream and the lymph flow; over respiration and digestion; over nutrition and disintegration; above all with its power of originating thought and establishing consciousness —if we think of this one system, of its life, of its nutrition, of its varied powers, and of its work—we stand still with sheer wonder, and almost with a denial on our lips that such a system can possibly be, or, being, can possibly do the work which has been assigned to it in the creative order. The human nervous system is more wonderful than suns and stars: it is the greatest miracle known to man.

But the nervous system is a mere part of the human organism. What a wonder is the nutritional system; and still more the reproductive! The mere minuteness of the cellular and molecular elements of which these and all the systems of the human body are composed is a standing amazement That these infinitesimal parts should have their definite and practically unvarying structure; that they should live; that each of them should do its daily work, and, working in concert with all the rest, should produce such astonishing results; that they should have the power of reproducing themselves, and of impressing upon their offspring to the remotest generations, their own physical structure, and their own power of function—this is all incomparably marvellous, and not to be reached in the full and comprehensive expression of it by the loftiest imaginings of the poet, or by the profoundest and most intelligent description of the philosophical writer.

The doctor's daily work lies among all these wonders; among the most delicate, most minute, most finished works of living art the world has ever seen. No artist has ever equalled the designer and constructor of man, and no art has ever been comparable with the design and the completed composition. The half-instructed doctor may not be conscious of the rare and delicate nature of the work he is called upon to perform, as the accredited regulator of this wonderful living mechanism; but the instructed physician and surgeon, the histologist, and the modern pathologist, look upon the wonders they presume to manipulate with ever increasing reverence, ever-increasing awe; and at times they approach their more difficult tasks with an inward "fear and trembling" which move the deepest depths of their being. What then can an honest and reverent medical scientist feel when some blatant blockhead rushes into the midst of all these wonders with his crude and barbarous compound, just as the savage with his war-paint and his assegai would rush upon the noblest works of civilization and art, and hurl them to the ground in the mere wantonness of ignorance and rage? What does the quack care? He cannot care because he does not know. Even the quack, the man who is resolved to be rich at any cost, might well give himself pause if he could but see for a single moment the wonders which are exposed in all their wondrousness to the instructed physiologist's hourly and constant view.

*Ars longa.* It cannot be otherwise. The physician's task, like the moralist's and the poet's, will never be completed. His first business is to lay all his foundations in absolute and demonstrated knowledge. That he is now doing with a thoroughness, and a continuous retrospection which have never been surpassed. Without this actual and real knowledge, this knowledge of structure and of function, of the actual and natural processes of the various parts in health, and of the actual and non-natural processes which constitute or produce disease—how can he proceed by honest and certain methods to modify such processes for the purposes of cure? Not only must the physician know the human organism in all its details of complicated structure and of normal and abnormal functions, but he must know, and with an equal precision, the thousand-and-one vegetable and mineral, natural and artificially produced substances which modify that function, and these too he must know in every detail of their nature and manifold effects. A truly prodigious task! A task demanding as high and almost higher powers of industry, intellect, and nobility of character than the world has ever yet seen. The world often sympathises with the pretender, and looks coldly upon the man of profound science and capacity. The world does this because it is ignorant. When it is better instructed it will send the pretender to gaol, and build the man of honest science "a monument more enduring than brass."

